# Explainable machine learning for early prediction of sepsis in traumatic brain injury: A discovery and validation study

**DOI:** 10.1371/journal.pone.0313132

**Published:** 2024-11-11

**Authors:** Wenchi Liu, Xing Yu, Jinhong Chen, Weizhi Chen, Qiaoyi Wu

**Affiliations:** 1 Department of Trauma Center/Emergency Surgery, The First Affiliated Hospital, Fujian Medical University, Fuzhou, China; 2 Department of Trauma Center/Emergency Surgery, National Regional Medical Center, Binhai Campus of the First Affiliated Hospital, Fujian Medical University, Fuzhou, China; 3 Department of Geriatrics, The First Affiliated Hospital, Fujian Medical University, Fuzhou, China; 4 Department of Geriatrics, National Regional Medical Center, Binhai Campus of The First Affiliated Hospital, Fujian Medical University, Fuzhou, China; Tehran University of Medical Sciences, ISLAMIC REPUBLIC OF IRAN

## Abstract

**Background:**

People with traumatic brain injury (TBI) are at high risk for infection and sepsis. The aim of the study was to develop and validate an explainable machine learning(ML) model based on clinical features for early prediction of the risk of sepsis in TBI patients.

**Methods:**

We enrolled all patients with TBI in the Medical Information Mart for Intensive Care IV database from 2008 to 2019. All patients were randomly divided into a training set (70%) and a test set (30%). The univariate and multivariate regression analyses were used for feature selection. Six ML methods were applied to develop the model. The predictive performance of different models were determined based on the area under the curve (AUC) and calibration curves in the test cohort. In addition, we selected the eICU Collaborative Research Database version 1.2 as the external validation dataset. Finally, we used the Shapley additive interpretation to account for the effects of features attributed to the model.

**Results:**

Of the 1555 patients enrolled in the final cohort, 834 (53.6%) patients developed sepsis after TBI. Six variables were associated with concomitant sepsis and were used to develop ML models. Of the 6 models constructed, the Extreme Gradient Boosting (XGB) model achieved the best performance with an AUC of 0.807 and an accuracy of 74.5% in the internal validation cohort, and an AUC of 0.762 for the external validation. Feature importance analysis revealed that use mechanical ventilation, SAPSII score, use intravenous pressors, blood transfusion on admission, history of diabetes, and presence of post-stroke sequelae were the top six most influential features of the XGB model.

**Conclusion:**

As shown in the study, the ML model could be used to predict the occurrence of sepsis in patients with TBI in the intensive care unit.

## Introduction

Traumatic brain injury (TBI) has presented a significant health challenge, characterized by high morbidity, disability, and mortality rates. Globally, over 10 million individuals have suffered from various forms and severities of TBI annually [1], imposing a considerable burden on public health systems [2,3]. Notably, sepsis, a highly complex and heterogeneous condition, has often led to substantial morbidity and mortality [4,5]. Researches have indicated that TBI patients were particularly vulnerable to infections, with an alarmingly high infection rate of up to 50%. Furthermore, sepsis accounted for a significant proportion (41–75%) of post-TBI infections and was a leading cause of hospital deaths in this patient population [3,6].

The early identification of TBI patients at risk of sepsis was crucial for reducing mortality and disease burden. However, a significant gap existed in the ability to accurately predict sepsis in this context. TBI patients admitted to the intensive care unit (ICU) often remained chronically unconscious, unable to provide immediate feedback on their condition. This limitation underscored the need for robust predictive tools capable of detecting the insidious development of infection and sepsis in these patients. Although several scoring systems [7–9], such as the Bedside Index of Severity in Acute Pancreatitis (BISAP) score, the systemic inflammatory response syndrome (SIRS) score, and the sepsis-related organ failure assessment (SOFA) score, were utilized to predict the severity and prognosis of TBI and sepsis, their performance in predicting sepsis was observed to be limited [10]. This highlighted a significant unmet need for more accurate and reliable predictive methods.

Machine learning (ML), a rapidly advancing field within artificial intelligence, offered distinct advantages over traditional statistical methods, including improved clinical prediction accuracy, enhanced performance, and faster processing speeds [11,12]. ML has been increasingly applied in medicine, facilitating early prediction and diagnosis of various diseases through data analysis and pattern recognition, thereby enhancing the efficacy of medical treatments and improving patient outcomes [11,13]. However, its application in predicting the risk of sepsis in TBI patients remained largely unexplored, representing a significant gap in current knowledge.

To address this gap, the study aimed to develop and validate six ML models based on clinical characteristics for the early prediction of sepsis risk in TBI patients. Furthermore, we sought to identify the best-performing model and interpret its predictions using the Shapley additive interpretation (SHAP) method. By leveraging the power of ML, we hoped to provide clinicians with a valuable tool for proactive sepsis management in this vulnerable patient population.

## Materials and methods

### Data source

The data for this retrospective cohort study was obtained from the Medical Information Mart for Intensive Care (MIMIC)-IV version 1.0 database [14], a comprehensive and high-quality repository of patient data from Beth lsrael Deaconess Medical Center between 2008 and 2019. MIMIC-IV consists of various clinically relevant data, such as medical records, medication administration, laboratory results, patient demographics, and disease codes based on the International Classification of Diseases. Additionally, we selected the eICU Collaborative Research Database (eICU-CRD)version 1.2 [15]as the external validation dataset. The eICU-CRD is a comprehensive andeasily accessible database that contains deidentified, detailed health data from over 200,000 ICU admissions across various medical centers in the United States from 2014 to 2015. Access and usage permissions for the two database were granted to author WCL after completing relevant online courses and passing a certification exam(Certification No: 46644825). Ethical approval for the use of this database in research was granted by the institutional review boards of the Massachusetts Institute of Technology (Protocol No:0403000206) and Beth lsrael Deaconess Medical Center (Protocol No:2001-P-001699/14). As the health information of patients in the database is uncertain, individual patient consent is not required.

### Study population

We developed a predictive model using clinical data to forecast the occurrence of sepsis in patients with TBI during their initial admission to the ICU in a retrospective analysis. The study included adult patients (age>18 years old) who were admitted to the ICU for the first time and had a confirmed diagnosis of TBI. The diagnosis of patients with TBI was determined by extracting the International Classification of Diseases (ICD) codes (ninth edition, code 85 or 10th edition, code S06) from the discharge diagnosis. Exclusion criteria encompassed non-first admissions, ICU stays <48 hours, repeat ICU admissions, and data missing >30%. The primary outcome measure for the clinical predictive model was the development of sepsis. Sepsis was diagnosed using the sepsis-3 criteria [16]. Specifically, patients who had a documented or suspected infection and an acute increase of≥2 points in their total SOFA score were classified as having sepsis.

### Data extraction and variable selection

Data extraction was performed from the MIMIC-IV database using pgAdmin PostgreSQL tools (version 1.22.1). A total of 46 variables were included, covering demographic information, laboratory results, vital signs, complications, medication treatments and ICU admission details. The sample variables consisted of age, gender, weight, and height. The identified complications encompassed hypertension, diabetes, cerebrovascular disease, chronic pulmonary disease, liver disease, kidney disease, etc. The values of vital signs (heart rate, respiratory rate, and body temperature,SPO2) were computed from ICU records. Laboratory results included platelets, hemoglobin, sodium, potassium, calcium, chloride, bicarbonate, albumin, total bilirubin, creatinine, urea nitrogen, blood glucose, pondus hydrogenii and lactate. We also included the Simplified Acute Physiology Score II (SAPSII score).The SAPSII score, is a severity scoring system used to assess the severity of illness for patients admitted to the ICU. It is calculated based on a range of physiological measurements, comorbidities, and age, and provides a quantitative estimate of the risk of hospital mortality. Medication treatments encompassed mechanical ventilation, blood transfusion, and vasopressors. Missing variables were imputed using multiple imputation methods. All data were collected within the first 24 hours of ICU admission.

### Construction and performances assessment of the ML models

In this study, we employed six different ML algorithms to develop prediction models, including Logistic Regression (LR), k-Nearest Neighbors(KNN), Gradient Boosting Machine (GBM), Support Vector Machine (SVM), Neural Network (NNET), and Extreme Gradient Boosting (XGB). We used the "createDataPartition" function from the caret package to split the patients into training and test sets in a 7:3 ratio, ensuring a random distribution of outcome events in both cohorts. During the model building process, we employed internal validation to assess the stability of the prediction models in the training set. We used ten-fold cross-validation as the resampling method to search for the optimal hyper parameters. In each iteration, we trained the models using 9-folds and used the final fold for hyper parameter tuning. Finally, we evaluated the performance of each model by computing the Area Under the ROC Curve(AUC) and the Average Precision (AP) of the Precision-Recall (P-R) curve in the test set, as well as comparing the sensitivity, specificity, and accuracy of the models. In addition, to assess the clinical applicability, we performed a Decision Curve Analysis (DCA) to quantify the net benefits at different thresholds and evaluate the utility of the models.

We employed the SHAP method to enhance the interpretability of the final model. Firstly, SHAP summary plot was used to illustrate the impact of model features. Secondly, SHAP dependency plot was employed to analyze the degree of influence of each feature on the model output. Finally, SHAP force plot was utilized to visually depict the effect of key features on the final model for individual patients.

### Statistical analysis

Metric data following a normal distribution was described using mean±standard deviation and differences between the two groups are compared using t-tests. For metric data that deviates from a normal distribution, median and quartile was used as descriptive statistics, and between-group comparisons are conducted using rank-sum tests. Count data is presented as the number of cases and proportions(%), and between-group comparisons are performed using chi-squared tests. Univariate logistic regression was used to analyze the clinical factors associated with sepsis and multivariate Logistic regression was used to further screen the factors with statistically significant differences in the univariate analysis (P<0.05), take a step backwards approach. A P<0.05 is considered statistically significant. All analyses were performed using Python 3.9.12 (Python Software Foundation) and R (version 4.1.3).

## Results

### Baseline characteristics

**Fig 1:**
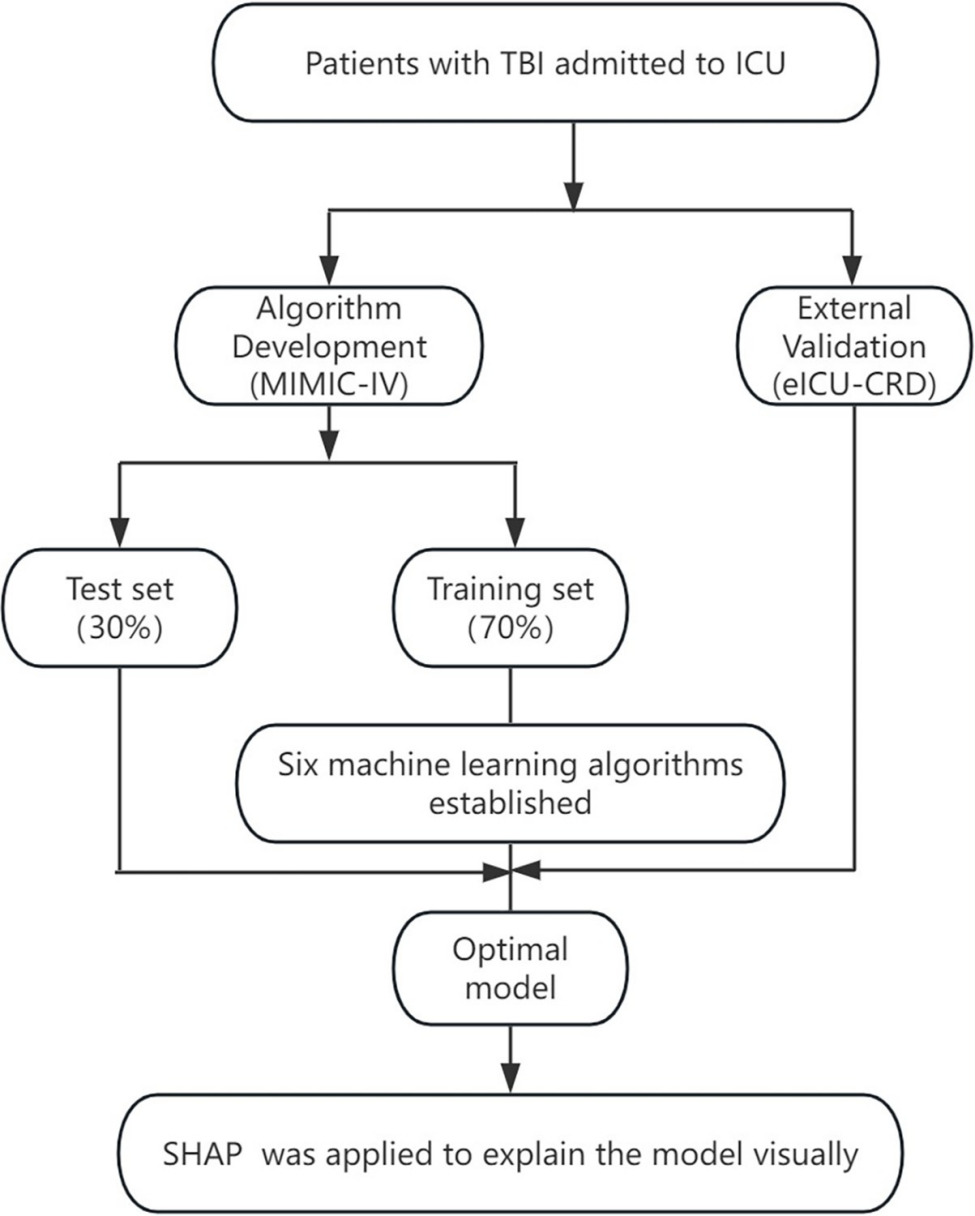
Analysis flow for the development and evaluation of models.

A total of 5167 TBI patients were identified from the database. After excluding patients with missing data (N = 1531), not admitted to ICU (N = 1890), diagnosed with sepsis before admission (N = 18), and less than 48h of hospitalization (N = 173), a total of 1555 patients (834 with secondary sepsis) were included, including 1089 in the training group and 466 in the verification group. The process of data extraction, preparatory training, and data testing using different ML algorithms was illustrated in Fig 1. In the training set, 584 cases of TBI patients (53.6%) were diagnosed with sepsis. Among the TBI sepsis patients in the training set, the average age was 68.33 (18.67) years, with 63.1% being male. Furthermore, 32.7% of TBI sepsis patients had diabetes, 31.5% used intravenous pressors, and 56.1% required mechanical ventilation. Table 1 provides a summary of the demographic and clinical data of the study cohort.

**Table 1:** Demographic and clinical characteristics of populations in the training and test sets.

Variables	Training set	Test set	P-value
N	1089	466	
Gender = Male (%)	687 (63.1)	293 (62.9)	0.983
Diabetes = Yes (%)	356 (32.7)	148 (31.8)	0.764
Hypertension = Yes (%)	636 (58.4)	277 (59.4)	0.745
Hyperlipidemia = Yes (%)	510 (46.8)	212 (45.5)	0.668
Myocardial_infarction = Yes (%)	192 (17.6)	84 (18.0)	0.909
Presence of post-stroke sequelae = Yes (%)	57 (5.2)	27 (5.8)	0.745
Cerebral_hemorrhage = Yes (%)	41 (3.8)	24 (5.2)	0.266
Coronary_artery disease = Yes (%)	320 (29.4)	139 (29.8)	0.908
Asthm = Yes (%)	111 (10.2)	45 (9.7)	0.818
Liver_disease = Yes (%)	58 (5.3)	31 (6.7)	0.362
AKI = Yes (%)	264 (24.2)	102 (21.9)	0.349
Oral immunosuppressant = Yes (%)	66 (6.1)	24 (5.2)	0.558
Use intravenous pressors = Yes (%)	343 (31.5)	141 (30.3)	0.935
Blood transfusion on admission = Yes (%)	266 (24.4)	119 (25.5)	0.689
Thrombocytopenia = Yes (%)	134 (12.3)	51 (10.9)	0.500
Bleeding in one area = Yes (%)	546 (50.1)	243 (52.1)	0.503
Bleeding position (%)			0.137
EDH	10 (0.9)	1 (0.2)	
SDH	151 (13.9)	85 (18.2)	
SAH	121 (11.1)	51 (10.9)	
IPH	276 (25.3)	111 (23.8)	
NS	531 (48.8)	218 (46.8)	
Bleeding in two or more areas = Yes (%)	543 (49.9)	223 (47.9)	0.503
Use mechanical ventilation = Yes (%)	611 (56.1)	264 (56.7)	0.886
Age (year)	72(57,83)	71(56,84)	0.871
Heart rate (beats/min)	87(75,96)	87(74,95)	0.697
Respiratory rate (/min)	19(16,21)	19(15,21)	0.216
Temperature (°C)	36.83(36.56,37.11)	36.83(36.49,37.06)	0.050
SPO2(%)	100(97,100)	100(97,100)	0.001
SAPSII score	35(28,40)	35(28,40)	0.350
GCS score	15(15,15)	15(15,15)	0.871
WBC (× 10^9^ /L)	9.1(6.6,12.4)	9.2(6.6,12.1)	0.998
Platelets(× 10^9^ /L)	216(169,270)	217(169,273)	0.973
Hemoglobin (g/dL)	12.7(11.4,14.0)	12.8(11.3,14.0)	0.980
RDW (%)	13.7(13.1,14.7)	13.8(13.1,14.8)	0.215
Hematocrit (%)	37.9(34.0,41.6)	38.2(34.3,41.7)	0.720
Total bilirubin(mg/dL)	0.5(0.4,0.9)	0.6(0.4,0.9)	0.136
Creatinine (mg/dL)	1.0(0.8,1.2)	0.9(0.8,1.2)	0.293
PT (s)	12.6(11.5,14.2)	12.7(11.5,14.5)	0.960
PTT (s)	28.2(25.2,32.0)	28.4(25.7,32.2)	0.269
INR	1.1(1.0,1.3)	1.1(1.0,1.3)	0.773
Urea nitrogen (mg/dL)	18(13,24)	17(12,25)	0.256
Glucose(mmol/L)	6.7(5.5,8.6)	6.7(5.5,8.5)	0.682
Sodium (mg/dL)	140(137,142)	139(137,141)	0.013
Potassium (mg/dL)	4,1(3.8,4.5)	4,2(3.8,4.6)	0.119
Calcium (mg/dL)	8.8(8.2,9.3)	8.8(8.3,9.3)	0.789
Chloride (mg/dL)	103(100,106)	102(99,105)	0.017
Bicarbonate (mEq/L)	25(22,27)	25(22,27)	0.864
Albumin(mmol/L)	3.8(3.3,4.2)	3.7(3.3,4.2)	0.191
Pondus Hydrogenii	7.39(7.32,7.43)	7.39(7.33,7.44)	0.125
Lactate (mmol/L)	1.8(1.3,2.7)	1.8(1.3,2.7)	0.217

AKI: Acute kidney injury; EDH: Extradural hemorrhage; SDH: Subdural bleeding;SAH: Subarachnoid hemorrhage;IPH: Intracerebral parenchymal hemorrhage;NS: Unknown;SAPSII score: Simplified Acute Physiology Score II; GCS score:Glasgow Coma Scale; WBC: White blood cell, RDW: Red blood cell width; PT: Prothrombin Time;PTT: Partial thromboplastin time; INR: International normalized ratio.

### Variable importance

The logistic regression was used to identify clinical predictive factors for the occurrence of sepsis in TBI patients. A total of 20 variables were statistically significant in univariate analysis (P <0.05). Further, variables with a significance level of P < 0.05 were included in a multivariable logistic regression model using a backward stepwise method. The results showed that the use of mechanical ventilation, SAPSII score, use intravenous pressors, blood transfusion on admission, history of diabetes, and presence of post-stroke sequelae were independent predictors of sepsis in TBI patients, as shown in Table 2.

**Table 2:** Univariate and multivariate logistic regression analyses.

Variables	Univariate OR (95% CI)	P-value	Multivariate OR (95% CI)	P-value
Gender(%)		0.014		0.294
Male	1.36 (1.07–1.75)		1.18 (0.87–1.59)	
Diabetes(%)		0.002		0.025
Yes	1.50 (1.16–1.95)		1.50 (1.05–2.14)	
Myocardial_infarction(%)		0.007		0.809
Yes	1.56 (1.13–2.14)		1.06 (0.67–1.68)	
Presence of post-stroke sequelae(%)		0.005		0.030
Yes	2.31 (1.28–4.17)		2.20 (1.08–4.49)	
Coronary artery disease(%)		0.007		0.624
Yes	1.44 (1.11–1.88)		1.10 (0.74–1.64)	
AKI(%)		0.020		0.230
Yes	1.40 (1.05–1.85)		1.26 (0.86–1.84)	
Use intravenous pressors(%)		<0.001		<0.001
Yes	3.52 (2.79–4.44)		1.63 (1.27–2.10)	
Blood transfusion on admission(%)		<0.001		<0.001
Yes	5.67 (4.03–7.98)		2.52 (1.65–3.84)	
Thrombocytopenia (%)		<0.001		
Yes	3.78 (2.44–5.84)		1.36 (0.79–2.35)	
Use mechanical ventilation(%)		<0.001		<0.001
Yes	7.74 (5.90–10.15)		5.19 (3.77–7.15)	
Heart rate	1.01 (1.00–1.01)	0.029	1.01 (1.00–1.02)	0.133
SAPSII score	1.03 (1.02–1.04)	<0.001	1.02 (1.00–1.03)	0.026
WBC	1.04 (1.01–1.06)	0.005	1.01 (0.98–1.04)	0.588
Platelets	1.00 (1.00–1.00)	0.020	1.00 (1.00–1.00)	0.279
PT	1.02 (1.00–1.04)	0.041	1.00 (0.98–1.02)	0.896
Glucose	1.00 (1.00–1.01)	0.001	1.00 (1.00–1.00)	0.616
Calcium	0.80 (0.69–0.92)	0.002	0.96 (0.79–1.17)	0.697
Chloride	1.02 (1.00–1.05)	0.032	1.01 (0.98–1.03)	0.702
Bicarbonate	0.95 (0.92–0.98)	<0.001	1.00 (0.96–1.04)	0.994
Albumin	0.61 (0.51–0.73)	<0.001	0.94 (0.72–1.22)	0.626

AKI: Acute kidney injury; SAPSII score:Simplified Acute Physiology Score II; WBC: White blood cell; PT: Prothrombin Time.

### Model comparisons

**Fig 2:**
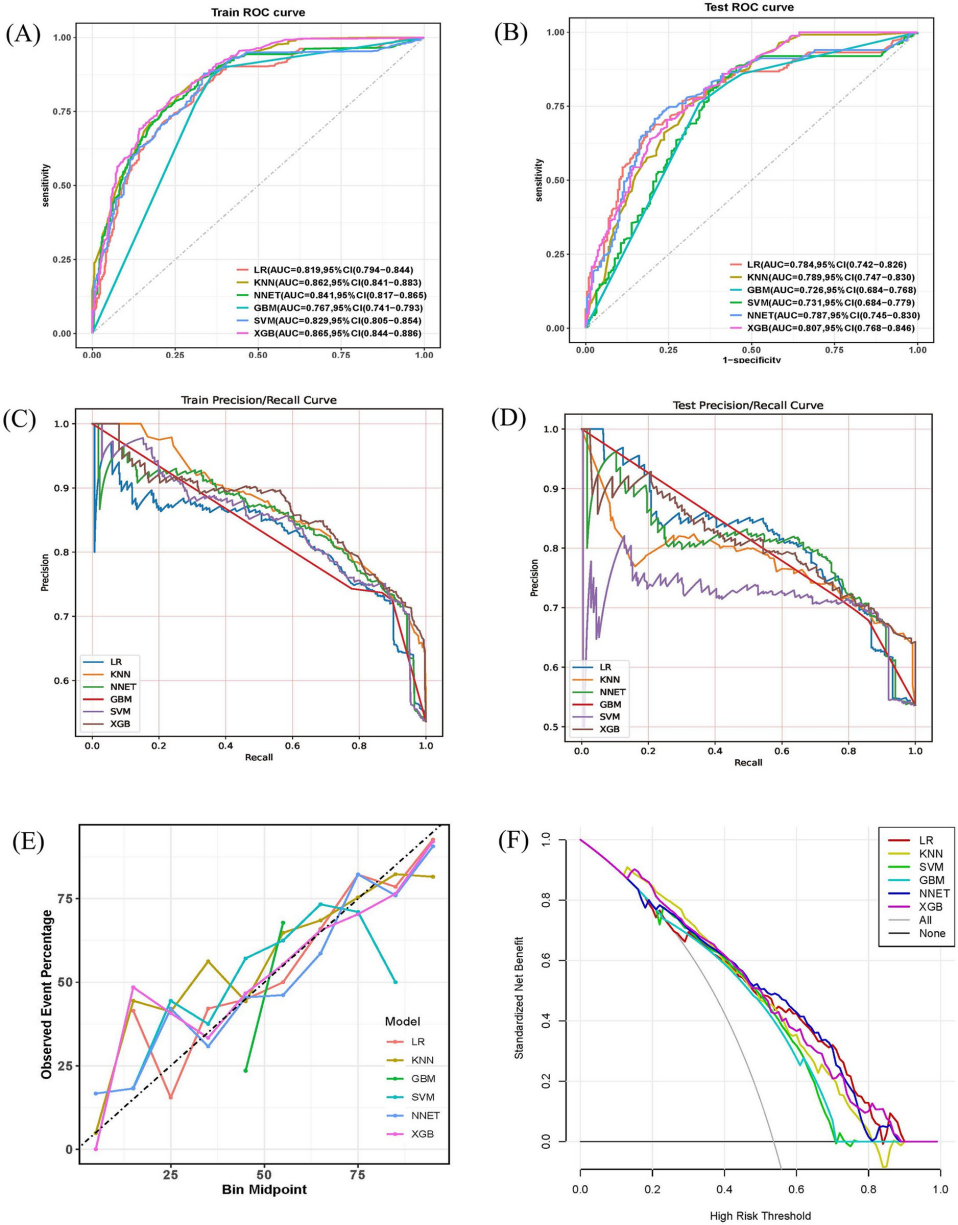
Construction and comparison of multiple ML models. Receiver operator characteristic (ROC) curves(A and B) and Precision-Recall (PR) Curves(C and D) of the six ML models in training set and test set. (E)The calibration curve of the ML models in test set. (F)The net benefit of the six ML models at different threshold probabilities for predicting sepsis in TBI patients.

**Fig 3:**
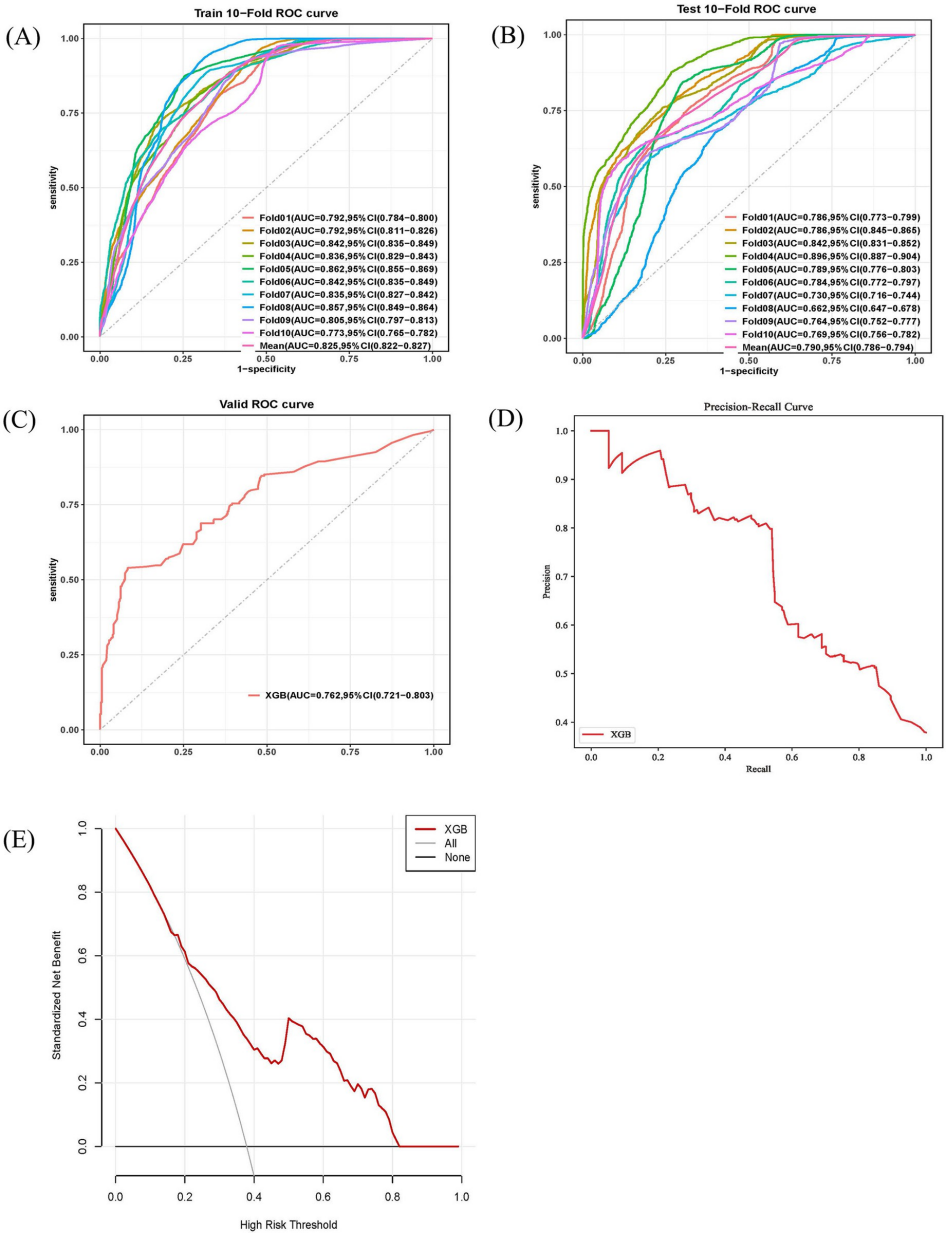
Assessment of XGB prediction model. The ROC curves of XGB using the 10-fold cross-validation on the training set (A) and testing set (B). The ROC curve (C), precision recall curve(D) and decision curve analysis (E) of the GBM model in the external validation data set.

Based on the predictive factors, six ML models including LRKNN, SVM, GBM, NNET, and XGB were constructed in the training set. The AUC values for the test set were 0.784,0.789,0.731, 0.726,0.787, and 0.807 respectively. Among them, the XGB model exhibited the strongest predictive ability with an AUC of 0.807 (95% C1: 0.768–0.846), while the GBM model had the lowest discriminative ability with an AUC of 0.726 (95% Cl:0.684–0.768) (Fig 2A and 2B). Similarly, the Precision-Recall curves also showed consistent results in the training and test sets (Fig 2C and 2D). In the calibration curve, the y-axis represents the actual probability of sepsis occurrence in the study cohort, while the x-axis represents the estimated probability by the models. As shown in Fig 2E, the XGB model demonstrated high consistency between the estimated and actual probabilities. The decision curve compared the net benefit of the best mode with other clinical decision methods. As shown in Fig 2F, the XGB model had a higher net benefit compared to other ML models, indicating its superiority in predicting sepsis in this cohort. A comparison of the predictive performance among different ML models was showed in Table 3. Fig 3 displays the ROC performance of the XGB model after10-fold cross-validation. The results indicate that the average AUC in the training set was 0.825 (0.822–0.827) (Fig 3A), and the average AUC in the test set was 0.790 (0.786–0.794) (Fig 3B), demonstrating the strong stability of the XGB model.

**Table 3:** Performance of the machine learning models.

**Training Sets**	**Models**	**AUC**	**Accuracy**	**Sensitivity**	**Specificity**	**Precision**	**F1 score**
	LR	0.819	0.757	0.818	0.685	0.750	0.783
	KNN	0.862	0.776	0.807	0.741	0.782	0.794
	SVM	0.829	0.779	0.878	0.663	0.751	0.810
	GBM	0.767	0.763	0.868	0.642	0.737	0.797
	NNET	0.841	0.769	0.750	0.790	0.805	0.777
	XGB	0.865	0.780	0.795	0.762	0.795	0.795
**Test Sets**	**Models**	**AUC**	**Accuracy**	**Sensitivity**	**Specificity**	**Precision**	**F1 score**
	LR	0.784	0.721	0.804	0.625	0.713	0.756
	KNN	0.789	0.723	0.748	0.694	0.739	0.744
	SVM	0.731	0.725	0.836	0.597	0.706	0.766
	GBM	0.726	0.710	0.828	0.574	0.692	0.754
	NNET	0.787	0.727	0.720	0.699	0.743	0.752
	XGB	0.807	0.745	0.752	0.773	0.786	0.748

LR: Logistic Regression; KNN: k-Nearest Neighbors;SVM: Support Vector Machine;GBM:Gradient Boosting Machine; NNET:Neural Network; XGB:Extreme Gradient Boosting; AUC: Area under the curve.

In the externally validated data set, a total of 602 patients were included, with 228 (37.8%) had TBI. The XGB model achieved an AUC of 0.762 (95%Cl 0.721–0.803), (Fig 3C). To assess the accuracy and clinical benefit of the model, precise recall curves (Fig 3D) and decision analysis curves (Fig 3E) were plotted. The model demonstrated good calibration in the external validation set, confirming its reliability. In conclusion, the XGB model, which exhibited clinical applicability, was identified as the best prediction model in this study.

### Visualization by SHAP

**Fig 4:**
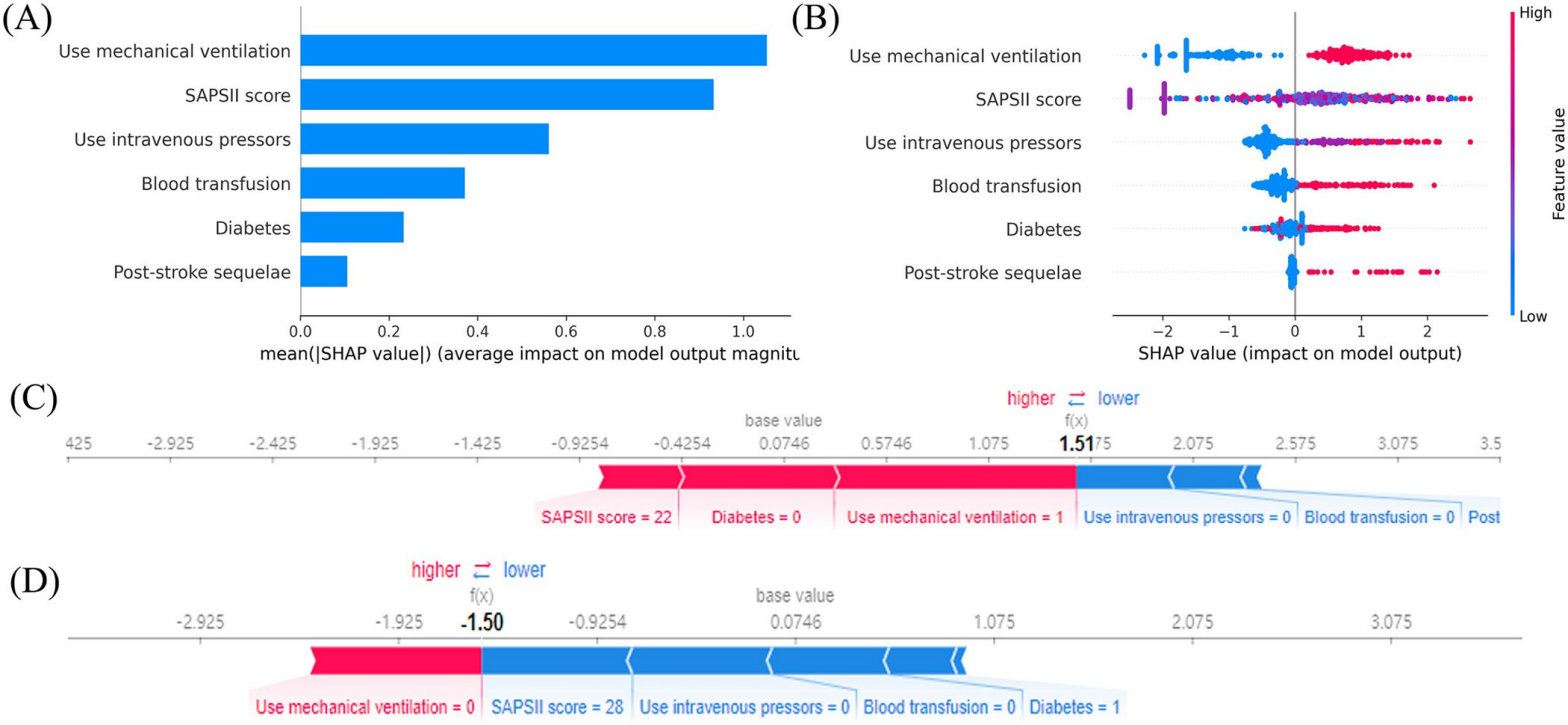
SHAP analysis result. (A) The top 6 variables ranked by the absolute SHAP values based on the mean importance; (B) Explanation of the 6 most important stable risk factors using the optimal model.(C and D) Model predictions by randomly drawing a single sample from the validation cohort.

Fig 4A presents the feature importance analysis of the TBI sepsis data. Fig 4B illustrates the distribution of SHAP values for each feature with the features ranked in descending order of importance.When the SHAP values positive, it signifies a positive contribution to the model’s prediction of sepsis. Conversely, a negative SHAP value indicates a negative contribution to the model’s prediction of sepsis. From Fig 4B, it can be observed that the use of mechanical ventilation has the greatest impact on the model’s predictions. Additionally, as the use of mechanical ventilation increases, the probability of the sample being predicted as septicemia increases. In other words, this feature has a positive influence on the prediction of sepsis, which aligns with clinical experience.

One individual predicted as a sepsis patient and another individual predicted as a non-sepsis patient were selected for individual factor analysis. In the selected representative cases, the individual with a higher SHAP value (1.51) was predicted to have a higher probability of developing sepsis(Fig 4C). On the other hand, the individual suggesting the need for preventive measures with a lower SHAP value (-1.5) was predicted to have a lower probability of being affected by the disease (Fig 4D).

## Discussion

In the study, an explainable ML model was successfully developed and validated for the early prediction of sepsis risk in patients with TBI. This novel approach holds significant implications for improving clinical decision-making and patient outcomes in the intensive care setting.

The findings underscore the utility of advanced ML techniques in analyzing complex clinical data to identify patients at high risk for sepsis. The XGB model, in particular, demonstrated robust predictive performance, achieving an AUC of 0.807 in internal validation and maintaining strong performance with an AUC of 0.762 in external validation. This suggested that the model has the potential to generalize well to different populations and clinical settings.

The utilization of the large-scale and high-quality MIMIC-IV database, which has been a cornerstone in numerous critical care studies, allowed us to extract a comprehensive dataset of TBI patients. This dataset served as a robust foundation for developing disease prediction models using various ML algorithms. By leveraging this rich data source, we were able to address a significant knowledge gap in sepsis prediction tools specifically tailored for TBI patients. In addition, the application of ML in early sepsis prediction following TBI represents a significant advancement in clinical decision support. Traditional methods of sepsis prediction often rely on manual monitoring and assessment, which can be time-consuming and subject to human error. By harnessing the power of ML, we can analyze vast amounts of patient data rapidly and accurately, enabling the timely identification of individuals at risk of sepsis. This approach not only enhances the efficiency of clinical workflows but also improves patient outcomes by facilitating early intervention. Moreover, there is a notable knowledge gap in understanding the complex interactions between TBI and sepsis. While previous studies have examined various biomarkers and clinical factors associated with sepsis development, the predictive accuracy of these models has been limited. The use of ML in this context allows for the exploration of more nuanced relationships between these variables, providing deeper insights into the pathophysiology of sepsis in TBI patients. The study aimed to bridge this knowledge gap by offering a robust and explainable ML model for early sepsis prediction. Furthermore, we also validated the predictive performance of the XGB model in a large eICU-CRD database from the United States. This validation process confirmed the robustness and reliability of our approach, further supporting its potential for widespread clinical application.

In the study, we identified six simple variables as independent predictors of sepsis in TBI patients, which are the use of mechanical ventilation, SAPSII score, administration of intravenous vasopressors, blood transfusion at admission, history of diabetes, and post-stroke sequelae. After severe TBI, the incidence of cerebral ischemia and hypoxia is greater than 90%. Early mechanical ventilation can rapidly and effectively correct hypoxemia, improve cerebral hypoxia, reduce the mortality and disability rates of TBI patients [17]. However, the process of mechanical ventilation treatment often leads to ventilator-associated pneumonia, prolonging hospitalization and ICU stays, increasing the use of antibiotics, subsequently raising the risk of secondary sepsis in TBI patients [18].

The SAPSII score is a established method to assess the severity of illness in ICU patients upon admission [19], widely used to guide clinical management of critically ill patients [20,21]. Kadziotka et al. showed that the higher the SAPSII score, the greater the risk of mortality in patients, providing high predictive value for the prognosis of critically ill patients [22]. In terms of prognostic prediction for septic shock patients, the efficacy of the SAPSII score surpasses that of the SOFA score [23]. This study indicates that TBI patients with higher SAPSII scores have an increased risk of developing sepsis. Therefore, in the clinical setting of the intensive care unit, continuous monitoring and assessment of SAPSII scores for TBI patients upon admission is crucial for early identification of patients with a high sepsis incidence rate, enabling timely formulation of corresponding clinical decisions.

In TBI patients who continue to exhibit hypotension and inadequate tissue perfusion despite adequate fluid resuscitation, the preferred treatment is the initiation of intravenous pressors therapy. Therefore, TBI patients requiring vasopressor agents often present with critical conditions, intrinsically uncorrectable tissue hypoperfusion, and a higher risk of sepsis. In clinical practice, for TBI patients requiring maintenance of hemodynamic stability, early intervention with vasopressor therapy is essential to reduce the risk of sepsis and mortality. Additionally, blood transfusion upon admission increases the risk of sepsis, which may be attributed to the early and immunosuppressive inflammation response in cases of acute severe trauma. The severity of this response is heightened when patients receive transfusions of blood products [24,25]. While certain treatments such as blood transfusions are frequently necessary, their use should be judicious.

Diabetes mellitus is a chronic disease characterized by abnormal elevation of blood sugar levels, with its incidence increasing year by year. This study utilized Logistic regression analysis to identify a history of diabetes as an independent risk factor for the development of sepsis in TBI patients after admission. This is attributed to the compromised immune system and weakened defense mechanisms in diabetic patients, as well as the promotion of bacterial growth and reproduction by high blood sugar and glycosuria, leading to reduced neutrophil phagocytic capacity [26]. Some researchers believe that diabetes can impair the function of both innate and adaptive immune response cells inpatients, rendering them unable to control the growth of microorganisms during infection, thereby further contributing to the progression of sepsis [27].

Existing studies have indicated that patients with post-stroke sequelae often experience pulmonary infections due to prolonged bed rest, compromised nutritional and immune function, decreased coughing and expectoration, reduced airway self-cleaning capability, and aspiration. When combined with TBI, this can further exacerbate the condition, impacting the quality of life and prognosis of the patients, and leading to an increased risk of sepsis [28]. Therefore heightened vigilance, timely diagnosis, and early intervention are essential for TBI patients with post-stroke sequelae to achieve the desired therapeutic outcomes.

This study has several strengths. Firstly, it is based on two large databases that span a long period from 2008 to 2019. Various modern ML methods were used to construct and validate prediction models. Secondly, the selected features were determined through stepwise backward analysis, increasing representativeness and accuracy. These six variables are easily accessible in clinical practice, enabling the model to be easily implemented in the real world. However, the study also has some limitations. Firstly, participants in our study were sourced exclusively from the MIMIC-IV database. Although the data was internally verified in the same center and externally verified using the eICU-CRD database, further external validation in prospective multi-center trials is warranted. Future research endeavors should aim to establish definitive correlations by enlisting multi-center population cohorts comprising a larger patient pool. Secondly, it is important to note that our study employed a retrospective design, which, despite its strengths in utilizing existing data, may have introduced inherent biases. Additionally, missing data within the dataset, particularly regarding the severity of TBI, represented a significant limitation that should be considered while interpreting our findings. Nonetheless, we believe the proposed model may contribute to a better understanding of the risk of sepsis in TBI patients.

In summary, our study highlighted the clinical utility of an explainable ML model for early prediction of sepsis risk in TBI patients. The XGB model, developed using six key clinical features, demonstrates good predictive performance in both internal and external validations. Importantly, this model not only provides accurate predictions but also offers insights into the most influential factors contributing to sepsis development in this patient population. By identifying high-risk patients early, clinicians can implement timely interventions to mitigate the risk of sepsis, potentially improving patient outcomes and reducing healthcare costs associated with this serious complication. Our findings underscored the value of integrating advanced ML techniques into clinical decision-making.

## Conclusion

In this study, we proposed a personalized predictive model based on ML for predicting the risk of sepsis in TBI patients upon admission to the ICU. Additionally, we found that the ML model based on the XGB algorithm outperformed traditional logistic regression, showing excellent predictive performance and providing valuable prediction information for clinical decision-making.

## Supporting information

S1 File (ZIP)
